# In vitro activities of *Acacia nilotica* (L.) Delile bark fractions against Oral Bacteria, Glucosyltransferase and as antioxidant

**DOI:** 10.1186/s12906-020-03147-4

**Published:** 2020-11-23

**Authors:** Ali Mahmoud Muddathir, Ebtihal Abdalla M. Mohieldin, Tohru Mitsunaga

**Affiliations:** 1 School of Molecular Life Science, Faculty of Applied Biological Science, Gifu University, 1-1 Yanagido, Gifu, 501-1193 Japan; 2 Department of Horticulture, Faculty of Agriculture, University of Khartoum, Khartoum North-Shambat, Sudan; 3 Faculty of Pharmacy, University of Science and Technology, Omdurman, Sudan

**Keywords:** *Streptococcus sobrinus*, *Porphyromonas gingivalis*, Glucosyltransferase enzyme, *Acacia nilotica*, ABTS radical scavenging

## Abstract

**Background:**

Dental caries and periodontal disease are the most common chronic infectious oral diseases in the world. *Acacia nilotica* was commonly known in Sudan as Garad or Sunt has a wide range of medicinal uses. In the present study, antibacterial activity of oral bacteria (*Streptococcus sobrinus* and *Porphyromonas gingivalis*), inhibitory activity against glucosyltransferase (GTF) enzyme and antioxidant activity were assayed for methanolic crude extract of *A. nilotica* bark and its fractions.

**Methods:**

Methanoilc crude extract of *A. nilotica* bark was applied to a Sephadex LH-20 column and eluted with methanol, aqueous methanol, and finally aqueous acetone to obtain four fractions (Fr1- Fr4). Furthermore, the crude extract and fractions were subjected to analytical high performance liquid chromatography (HPLC). The crude extract and its fractions were assayed for antibacterial activity against *S. sobrinus* and *P. gingivalis* using a microplate dilution assay method to determine the minimum inhibitory concentration (MIC) and minimum bactericidal concentration (MBC), as well as GTF inhibition and antioxidant activity using ABTS radical scavenging method.

**Results:**

Fractions (Fr1 and Fr2) exhibited MIC values of 0.3 mg/ml against the *P. gingivalis*. Additionally, Fr2 displayed MBC value of 1 mg/ml against two types of bacteria. Fr4 showed an especially potent GTF inhibitory activity with IC_50_ value of 3.9 μg/ml. Fr1 displayed the best antioxidant activity with IC_50_ value of 1.8 μg/ml. The main compound in Fr1 was identified as gallic acid, and Fr2 was mostly a mixture of gallic acid and methyl gallate.

**Conclusions:**

The results obtained in this study provide some scientific rationale and justify the use of this plant for the treatment of dental diseases in traditional medicine. *A. nilotica* bark, besides their antibacterial potentiality and GTF inhibitory activity, it may be used as adjuvant antioxidants in mouthwashes. Further studies in the future are required to identify the rest of the active compounds.

## Background

Dental caries is an irreversible localized infection that results in progressive tooth decay [1]. It’s a common type of dental disease associated with microorganisms present on the tooth surface in dental plaque. One of the main etiologic factors of dental caries is considered to be *Streptococcus sobrinus*, which belong to the gram-positive mutans streptococci group. The presence of *S. sobrinus* is relatively higher on the molars compared to the anterior teeth [1, 2]. Several epidemiological studies have shown that the prevalence of *S. sobrinus* is more closely associated with high caries activities [3]. *S. sobrinus* can colonize the tooth surface and initiate plaque formation by synthesizing water-insoluble glucan from sucrose by glucosyltransferases (GTF), resulting in a firm attachment to the tooth surface and form lactic and other organic acids by fermentation of various sugars in foods [4]. Different strategies for preventing dental caries caused by cariogenic bacteria, such as depression of the growth of streptococcus, inhibition of GTF activity, and hydrolysis of glucans by enzymes, have been developed [5]. Further accumulation of plaque around the gingival margin and subgingival region may lead to shifts in the balance of the microflora from mainly gram-positive bacteria to gram-negative bacteria, and an increased number of gram-negative anaerobic bacteria will cause the development of periodontal diseases [6].

Periodontal diseases are chronic inflammatory disorders of bacterial origin that affect tooth-supporting tissues [7]. Over 700 bacterial species that have been identified in the oral cavity [8], only a few are associated with periodontitis, including *Porphyromonas gingivalis* [9]. *P. gingivalis* is a gram-negative bacterium closely associated with chronic periodontitis. High numbers of *P. gingivalis*, together with other periodontopathogens, induce a host immune response, which in turn leads to a destructive inflammatory process.

Oxidative stress observed in a diseased periodontium could result directly from excess reactive oxygen species (ROS) activity or antioxidant deficiency or indirectly by creating a pro-inflammatory state. Some studies reported that the excess production of ROS resulted in damages of gingival tissues, periodontal ligament, and alveolar bone [10–12]. Therefore, the search for an antioxidant that could be used to control these diseases as polyphenolic compounds are likely candidates [13].

*Acacia nilotica* (L.) Delile sub *nilotica* (Leguminosae) is a tree found in the central and northern parts of Sudan and is known in Sudanese folk medicine by the common name ‘Garad or Sunt.’ The fruit and the stem bark are regarded as a tonic and astringent and are used internally to treat colds, bronchitis, pneumonia, diarrhea, and dysentery [14, 15]. Fractionation of *A. nilotica* leaves and bark showed the presence of phenols condensed tannin [16], gallic acid, (+) catechin, (−) epigallocatechin-7-g’allate, catechin derivatives [17, 18], ellagic acid, kaempferol, and quercetin [19]. Parallelly many researches demonstrated its wide array of pharmacological activities, such as anti- HIV-1 protease [20], antibacterial [21], antioxidant, anticarcinogenic [22, 23] and anti-inflammatory activities [24]. In Sudanese and Indian traditional medicine, gargle the decoction of the *A. nilotica* bark is used to strengthens teeth and eliminates toothache [25–27]. Even though there is a little investigation on the potential beneficial effects of *A. nilotica* bark on oral health. Thus, we evaluated the effects of *A. nilotica* bark methanolic extract and its fractions on the growth of two oral bacteria included *S. sobrinus* and *P. gingivalis*. We also investigated their GTF inhibitory activity and antioxidant functions for oral hygiene purposes.

## Methods

### Reagents

All materials purchased from (Wako-Japan) except p-iodonitrotetrazolium violet from (Sigma-Aldrich-Japan**).**

### Plant materials and extraction

*A. nilotica* bark was collected from Sennar State, Sudan in May 2013 and then authenticated by the University of Khartoum, Faculty of Forest. Voucher specimens (SD-SS-01) are deposited in the Horticultural Laboratory, Department of Horticulture, Faculty of Agriculture, University of Khartoum. *A. nilotica* bark was shade dried and powdered before being extracted with methanol for 12 h three times. The extracts were filtered through Whatman No. 2 filter paper, and the solvent was removed under vacuum using a rotary evaporator.

### Fractionation of *A. nilotica* bark

The crude extract (400 mg /2 ml of 50% methanol) was applied to a Sephadex LH-20 column. The column was eluted with methanol (250 ml), methanol-water (80:20, v/v; 150 ml, Fr1), methanol-water (50:50, v/v; 250 ml, Fr2), methanol-water (5:95, v/v; 100 ml, Fr3) and finally acetone-water (70:30, v/v; 250 ml, Fr4). The fractions (Fr1–4) were concentrated in vacuo (38 °C) and freeze-dried to give four powders with approximate weights of 34.2 mg, 35.7 mg, 44.8 mg, and 244.5 mg for Fr1, Fr2, Fr3, and Fr4 respectively. Furthermore, the crude extract and fractions were subjected to high performance liquid chromatography (HPLC) with reversed-phase column C_18_ (Sunniest 4.5 mm i.d. X 250 mm). The solvent system used was as follows: a gradient program for 65 min from 5 to 100% methanol in water with 0.05% TFA (Trifluoroacetic acid) at a flow rate of 1 ml/min, monitored at 280 nm.

### Antibacterial activity assay

Minimum inhibitory concentration **(**MIC) was determined by the broth dilution method [28]. *S. sobrinus* 6715 and *P. gingivalis* ATCC 33277 were cultured in brain heart infusion broth. For *P. gingivalis* broth supplemented with 0.5 μg/ml vitamin K_3_ and 5 μg/ml hemin. The samples were tested for antibacterial activity in sterile 96-well plates. The inoculums were prepared by diluting the broth culture to 10^6^cell/ml for *S. sobrinus* and 10^8^cell/ml for *P. gingivalis* approximately. The experiments were performed in triplicate. Chlorhexidine was included in the assay as a positive control. Then cultures were incubated 24 h for *S. sobrinus* and 72 h for *P. gingivalis* at 37 °C under anaerobic condition. Microbial growth was indicated by adding 50 μl of (0.2 mg/ml) p-iodonitrotetrazolium violet (INT) to culture and incubated at 37 °C for 2 h. The MIC was defined as the lowest concentration that inhibited the color change of INT [29]. For minimum bactericidal concentration (MBC), 10 μl from wells that showed no color change were transferred to 100 μl of fresh media and then incubated for 24 h under the anaerobic condition at 37 °C. Microbial growth was also indicated by adding INT to culture. The MBC was defined as the lowest concentration that inhibited the color change of INT.

### Preparation of glucosyltransferase (GTF)

*S. sobrinus* 6715 was grown for 20 h at 37 °C in 4 L of Todd Hewitt broth. After centrifugation of the culture at 1300 *g* for 10 min at 4 °C, the cells were collected and then extracted with 8 M urea for 1 h with stirring. The crude enzyme solution containing urea was dialyzed against 10 mM sodium phosphate buffer (pH 6) until the urea was removed entirely. One milliliter of the crude enzyme solution was pipetted into a microtube and stored in a freezer at − 80 °C [30].

### GTF inhibitory activity assay

Insoluble glucan synthesized by GTF was measured turbidimetrically. GTF was incubated in 300 μl of 0.1 M phosphate buffer (pH 6.0) containing 1% sucrose, 0.5% dextran T-10, and in the presence or absence of samples at 37 °C for 3 h. The volume of the crude GTF solution used in the assay was determined by absorbance of around 1.0 at 590 nm. Chlorhexidine was used as a positive control [30]. The inhibition rate is expressed by the following equation: Inhibition (%) = 100 × (Ac – As)/Ac.

Where.

Ac: Absorbance of the control.

As: Absorbance of the sample.

### ABTS radical scavenging activity assay

2,2′-azino-bis (3-ethylbenzothiazoline-6-sulfonic acid diammonium salt) was dissolved in water to make a concentration of 7 mM. ABTS^+^ was produced by reacting to the ABTS stock solution with 2.45 mM potassium persulfate and allowing the mixture to stand in the dark at room temperature for 12–16 h before use. For the study of samples, the ABTS^+^ was diluted with phosphate-buffered saline 5 mM, pH 7.4 to obtain an absorbance of 0.70 at 734 nm. After the addition of 980 μl of diluted ABTS to 20 μl of samples, the absorbance reading was taken five minutes after the initial mixing [31]. Trolox was used as a positive control. The activity was measured as follows:

% ABTS scavenging activity =.

[(control absorbance – sample absorbance)/ (control absorbance)] × 100.

### Statistical analysis

The inhibitory activity of GTFs and antioxidant activities were expressed as the mean (mean ± standard deviation) value. The significant differences between samples were assessed by one-way analysis of variance (ANOVA) followed by pairwise comparison of the mean using Tukey’s multiple comparison test. Values were determined to be significant when *p* was less than 0.05 (*p<*0.05).

## Results

### Chromatogram data

**Fig. 1:**
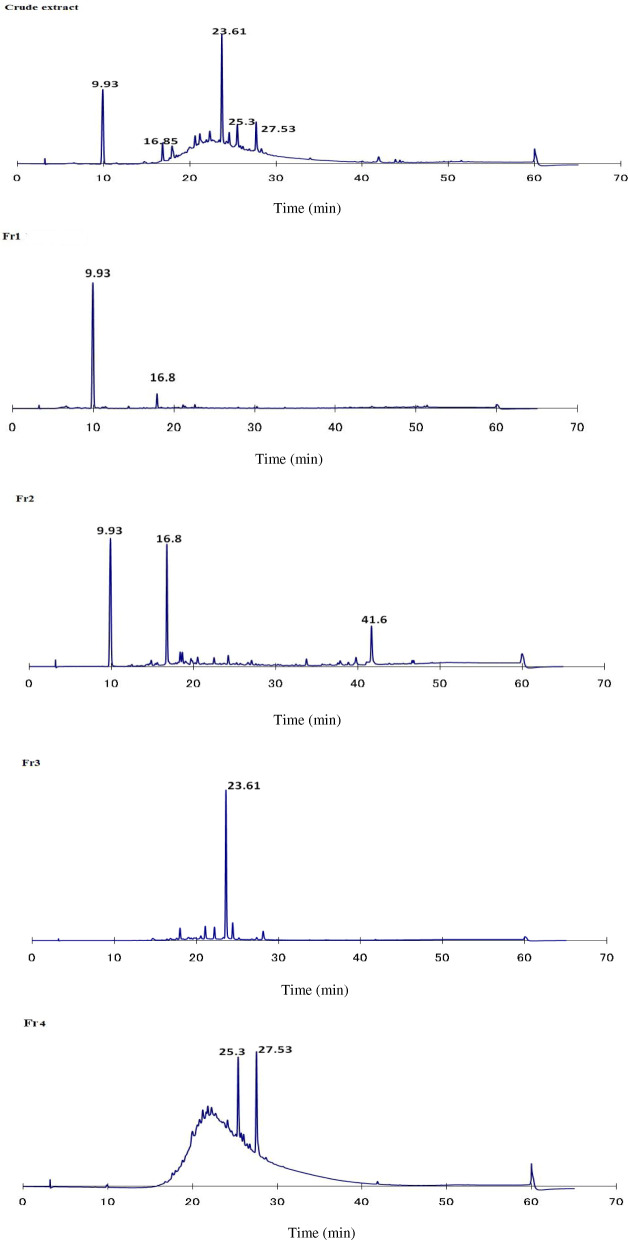
HPLC chromatogram of *Acacia nilotica* crude extract and its fractions (F1-F4)

**Fig. 2:**
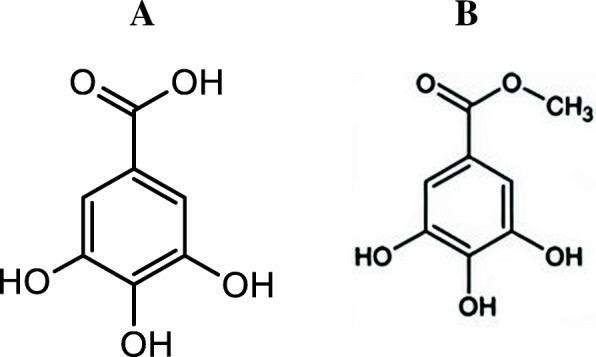
Molecular structure of gallic acid and methyll gallate. A: Gallic acid. B: Methyll gallate

**Fig. 3:**
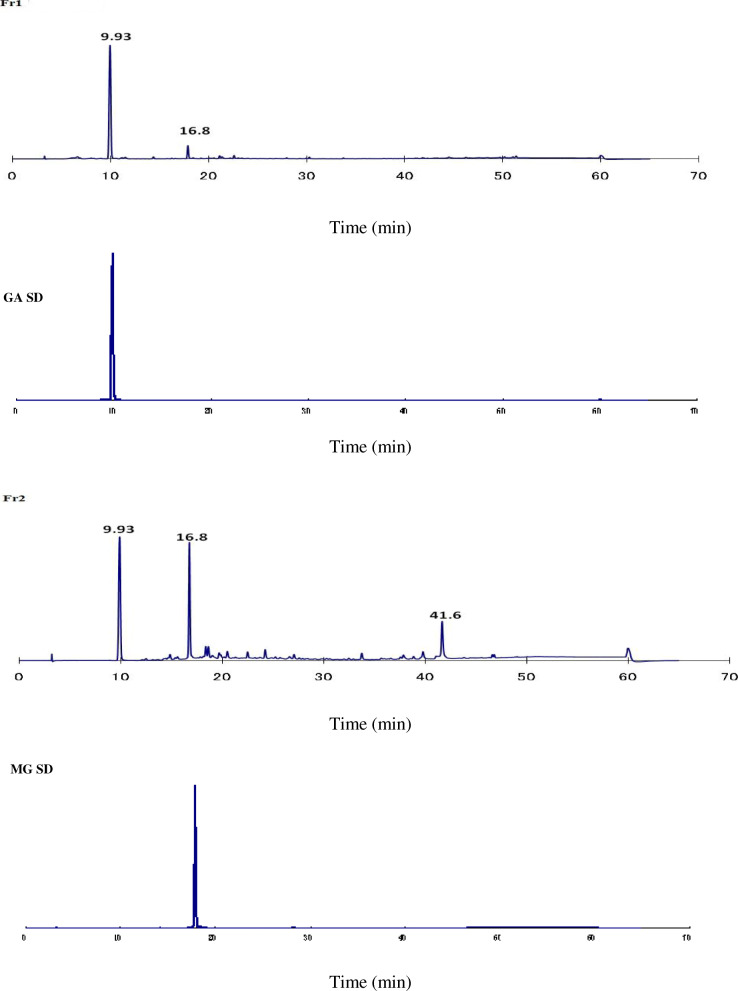
HPLC chromatogram of isolated fractions from *A. nilotica* (F1 and F2) and commercial standard (GASD: Gallic acid standard; MGSD: Methyl gallate standard)

Typical chromatograms of crude methanolic extract and fractions are shown in Fig. 1. The chromatographic peaks of Fr1 and Fr2 were identified according to a commercial standard of gallic acid (GA) and methyl gallate (MG) (Figs. 2, 3). Therefore, the Fr1 contained a GA at the retention time 9.9 min as the main compound. Fr2 contained a mixture of GA and MG at the retention time 9.9 and 16.8 min, respectively, in addition to unknown compound at the retention time of 41.6 min. In contrast, compounds in the other fractions are still unidentified.

### Antibacterial activity

In this study, the antibacterial MIC and MBC values of the *A. nilotica* bark and it’s fractions against *S. sobrinus* and *P. gingivalis* using dilution methods were presented in Table 1. The MIC values of 0.5 mg/ml or less were considered as good antibacterial activity. Generally, Fr2, Fr1 and crude extract demonstrated good MIC values against *S. sobrinus* and *P. gingivalis*. MIC value of 0.3 mg/ml was observed when Fr1 and Fr2 were used against *P. gingivalis*. Fr2 showed the best antibacterial activity against the two types of bacteria. Positive control demonstrated the most potent inhibition activity against the two types of bacteria.

**Table 1:** Antibacterial activities of *Acacia nilotica* bark and their fractions against *Streptococcus sobrinus* and *Porphyromonas gingivalis*

Samples	*Streptococcus sobrinus*		*Porphyromonas gingivalis*	
	MIC mg/ml	MBC mg/ml	MIC mg/ml	MBC mg/ml
Crude extract	0.5	2	0.5	2
Fr1	0.5	-^a^	0.3	4
Fr2	0.5	1	0.3	1
Fr3	1	–	0.5	–
Fr4	0.5	–	1	–
Chlorhexidine ^b^	0.0004	0.0004	0.0004	0.0004

^a^MBC was not detected

^b^Positive control

### GTF inhibitory activity

Fraction four (Fr4), methanolic crude extracts of *A. nilotica* bark, and Fr3 at the concentration of 100 μg/ml inhibited GTF secreted from *S. sobrinus* to a remarkable extent with more than 85% inhibitions (Table 2). Judging from the IC_50_ values; Fr4, and methanolic crude extracts showed remarkable inhibition of GTF activity with IC_50_ values of 3.9 and 8.4 μg/ml, respectively (Table 2). Chlorhexidine, as a positive control, showed good activity against GTF.

**Table 2:** The inhibitory activities of *Acacia nilotica* bark and their fractions against glucosyEltransferase (GTFs) enzyme

Samples	Inhibitory activity (%) at 100 μg/ml	IC_50_ μg/ml
Crude extract	90.1 ± 1.53^a,b^	08.4 ± 3.8^a^
Fr1	02.57 ± 3.57^d^	> 100
Fr2	59.7 ± 4.76^c^	89.4 ± 4.1^c^
Fr3	85.7 ± 0.85^b^	35.4 ± 19.4^b^
Fr4	92.8 ± 0.71^a^	03.9 ± 2.7^a^
Chlorhexidine ^*^	90.2 ± 0.12^a,b^	05.7 ± 4.2^a^

Means with different letters in the same column were significantly different at the level (*p* < 0.05); *n* = 3

* Positive control

### ABTS radical scavenging activity

**Fig. 4:**
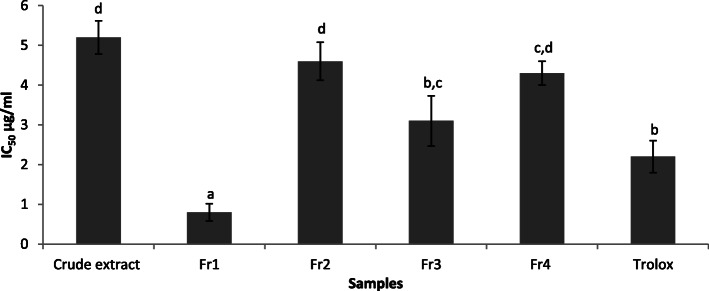
Free radical scavenging properties of *A. nilotica* bark and its fractions by using ABTS photometric assay. Values not followed by a common letter were significantly different at the level (*p* < 0.05)

IC_50_ value is the effective concentration at which antioxidant activity is 50%. The ABTS radical scavenging activity (IC_50_) of crude extracts and fractions were ranged between 0.8–5.2 μg/ml (Fig. 4). The results also revealed that Fr1 had a significant antioxidant activity with an IC_50_ value of 0.8 μg/ml compared to the trolox as a positive control (2.1 μg/ml). As described previously, the gallic acid was the main compound in this Fraction. Fraction three (Fr3) also demonstrated an excellent antioxidant activity in which there were no significant differences between Fr3 (3.1 μg/ml) and positive control.

## Discussion

### Chromatogram data

HPLC analysis is the best way for the chemical profiling of plant extract. The described method showed relatively good separation of specific fractions. Identification of phenolic compounds in methanolic extract of *A. nilotica* bark was carried out by using the available standards (GA and MG) as shown in Fig. 3. In the present study, Fr1 and F2 contained a GA and mixture GA and MG respectively. These results were in agreement with Sharma et al. [32] and Leela et al. [33]. They isolated and purified GA and MGfrom *A. nilotica* bark through open column chromatography and HPTLC. In contrast, Sadiqa et al. [34] reported that quantification of bark extract of *A. nilotica* collected from Pakistan by using HPLC/DAD showed that catechin, isoquercetin, tannic acid and quercetin contained in considerable quantities. However, the gallic acid was found in leaves and pods only. Also, Zhang and Lin [35] stated that the broad peaks in HPLC chromatogram spectra indicated that there was a large structural heterogeneity with different degrees of polymerization. Fr4 HPLC chromatogram contains a broad peak after 17 min retention time (Fig. 1). This broad peak was suggested to be a mixture of higher molecular weight of proanthocyanidins (condensed tannins).

### Antibacterial properties

Several authors suggested that the effective antimicrobial agents from herbal extracts along with their derived materials against cariogenic and periodontopathic bacteria, could form an important part preventing dental caries and periodontal diseases [36, 37]. Gibbons [38] suggested that isolated phytochemicals should have MIC< 1 mg/ml, therefore in this study MIC value of 0.5 mg/ml or less for *S. sobrinus* and *P. gingivalis* was considered as a good antibacterial. Even though gram-negative bacteria are more resistant than gram-positive due to the presence of outer membrane, which acts as a barrier to environmental substances, including antibiotics [39, 40]. The observed activity of Fr1 and Fr2 against gram-negative bacteria (*P. gingivalis*) could be explained by the fact that the active compounds might act by inhibiting the bacterial growth without necessarily penetrating into the bacterial cell itself [41]. In earlier studies, extracts of the *A. nilotica* pods displayed antibacterial activity against *Staphylococcus aureus* with MIC value of 0.4 mg/ml. Abdel Nabi et al. [21] suggested that the antimicrobial activity of *A. nilotica* fruit extract (MIC = 0.05–1.6 mg/ml) was not due to tannins but to another substance(s). *A. nilotica* bark and leaves showed good antibacterial activities against some gram-positive and gram-negative bacteria [42–44]. Furthermore, *A. nilotica* bark has been shown to contain simple phenolic compounds like GA and MG, which have often been implicated in antibacterial activity. Deshpande and Kadam [45] reported that ethanol extract of *A. nilotica* bark showed activity against *S. mutans* with MIC value of 5 mg/ml. The results obtained in this study are similar to Kang et al. [46], who reported that both GA and MG had inhibitory effects on the growth of cariogenic (MIC = 8 mg/ml) and periodontopathic bacteria (MIC = 1 mg/ml). Hence, MG and GA might be used to prevent the formation of oral biofilms. However, MG was more effective in inhibiting bacterial growth and formation of *S. mutans* biofilm than GA.

### Enzyme inhibitory activity

Mutans streptococci group colonize at the tooth enamel surface and initiate plaque formation by their ability to make extracelluar polysaccharides from sucrose using glucosyltransferase which is a major component in the development of dental caries [47]. The previous report mentioned that methanolic extracts of *A. nilotica* bark contained a considerable amount of condensed tannins [48]. The HPLC chromatogram of F4 indicated the presence of condensed tannins. This type of tannin is available in barks, oolong tea, green tea and grape seed, which are potent GTF inhibitors owing to their polyphenolic moiety being able to form hydrogen bonds with GTF [5]. Besides, high molecular weight plant polyphenols displaying strong anti-GTF activity have a common structural feature shared with catechin-based oligomeric forms (condensed tannins) [49]. Chlorhexidine showed potent inhibitory activities against two types of bacteria and GTF enzyme. However, it has been reported that chlorhexidine is cytotoxic to human periodontal ligament cells, inhibits protein synthesis, and affects mitochondrial activity, thus having detrimental effects on vital tissues [50, 51].

### Antioxidant activity

The role of antioxidants is to remove harmful oxidants or (ROS) as soon as they form or to repair the damage caused by ROS. Numerous antioxidants have been tried and tested both by systemic administration and as mouthwashes. These include synthetic products like vitamins to natural products like wine and green tea [52, 53]. Additionally, few studies were carried out on the antioxidant activity of oral hygiene products. Battino et al. [54] showed that a few antioxidant-enriched toothpastes displayed a clear antioxidant activity. In vivo assay, Tamaki et al. [55] stated that the oral administration of resveratrol improves oxidative stress and prevents the progression of periodontitis in a rat periodontitis model. In this study, the antioxidant assay is based on the ability of an antioxidant to scavenge ABTS radicals. It is a simple, rapid and inexpensive antioxidant assay usually used for the evaluation of antioxidant capacity [56]. The ABTS radical is soluble in water and organic solvents, enabling the determination of the antioxidant capacity of both hydrophilic and lipophilic samples [57]. The finding in this study agreed with Sohi et al. [58]. They stated that pretreatment of peripheral blood lymphocytes with gallic acid effectively inhibit lipid peroxidation and apoptosis induced by oxidative stress and it was found to be a stronger antiradical than trolox.

Furthermore, our results support the reports of Sultana et al.; Singh and Arora [59, 60] who reported that *A. niloica* bark extracts showed a strong antioxidant activity by using DPPH radical scavenging activity assay. Houde et al. [13] demonstrated that proanthocyanidins contained in grape seed extracts were potent antioxidant properties and should be considered a potential agent in the prevention of periodontal diseases. So, it is not surprising that our extract, which is rich in polyphenolic compounds, possessed high antioxidant activity and it could be used for oral hygiene.

Therefore in the present study, we showed that the crude extract of *A. nilotica* and their fractions can affect bacterial and GTF enzyme viability. Furthermore, some fractions showed significant antioxidant activity. The might be proposed mechanisms of action to explain the crude extract or their fractions antimicrobial activities, including direct action on microbial metabolism through inhibition of oxidative phosphorylation or inhibition of extracellular microbial enzymes required for microbial growth. Another overlooked advantage of GTF-targeting inhibitors is their potential to possess anti-biofilm activity without being (with less) bactericidal, thus providing alternative methods to prevent biofilm-related diseases with minimal effect on the ecological balance in the microbial community [61]. Also, Jeon et al. [62] reported that caries antagonistic activity of antioxidants polyphenols had been attributed to three main mechanisms (i) Direct antimicrobial effect by killing the responsible bacteria, (ii) Inhibition of extracellular polysaccharide production, and (iii) Impairment of bacterial adhesion.

## Conclusions

The results of the present study suggest that besides antibacterial potentiality and GTF inhibitory activity of *A. nilotica* bark may be used as adjuvant antioxidants in mouthwashes and may provide treatment strategies for periodontal diseases in the future. However, additional tests including identify the rest of the active compounds, cytotoxicity assay, experimental models and the pharmacological applicability are required soon.

## Data Availability

The data sets that support the conclusions of this article are included in the article.

## Abbreviations

GTF: Glucosyltransferases; ROS: reactive oxygen species
Fr: Fractions
HPLC: High performance liquid chromatography
TFA: Trifluoroacetic acid
MIC: Minimum inhibitory concentration
INT: P-iodonitrotetrazolium violet
MBC: Minimum bactericidal concentration
ABTS: 2,2′-azino-bis (3-ethylbenzothiazoline-6-sulfonic acid diammonium salt)
ANOVA: Analysis of variance
GA: Gallic acid
MG: Methyl gallate
DPPH: 1, 1-diphenyl-2-picrylhydrazyl
